# The Singularity Paramagnetic Peak of Bi_0.3_Sb_1.7_Te_3_ with *p*-type Surface State

**DOI:** 10.1186/s11671-021-03650-8

**Published:** 2022-01-15

**Authors:** Shiu-Ming Huang, Pin-Cing Wang, Pin-Cyuan Chen, Jai-Long Hong, Cheng-Maw Cheng, Hao-Lun Jian, You-Jhih Yan, Shih-Hsun Yu, Mitch M. C. Chou

**Affiliations:** 1 Department of Physics, National Sun Yat-Sen University, Kaohsiung, 80424 Taiwan; 2 National Synchrotron Radiation Research Center, Hsin-Chiu, 80076 Taiwan; 3 Department of Materials and Optoelectronic Science, National Sun Yat-Sen University, Kaohsiung, 80424 Taiwan; 4 Taiwan Consortium of Emergent Crystalline Materials, TCECM, National Sun Yat-Sen University, Kaohsiung, 80424 Taiwan; 5 Center of Crystal Research, National Sun Yat-Sen University, Kaohsiung, 80424 Taiwan

**Keywords:** Paramagnetic susceptibility peak, Topological material, Dirac point, Surface state

## Abstract

The magnetization measurement was performed in the Bi_0.3_Sb_1.7_Te_3_ single crystal. The magnetic susceptibility revealed a paramagnetic peak independent of the experimental temperature variation. It is speculated to be originated from the free-aligned spin texture at the Dirac point. The ARPES reveals that the Fermi level lies below the Dirac point. The Fermi wavevector extracted from the de Haas–van Alphen oscillation is consistent with the energy dispersion in the ARPES. Our experimental results support that the observed paramagnetic peak in the susceptibility curve does not originate from the free-aligned spin texture at the Dirac point.

## Introduction

Recently, the singularity paramagnetic peak in the susceptibility curve has been reported in topological materials [1–5]. The paramagnetic peaks were invariant to a temperature ranging from 2 to 300 K. It is qualitatively understood to be originating from the particular carrier spin texture in a topological surface state [6, 7]. The spin texture rotates in different clockwise directions for carriers above and below the Dirac point [8, 9]. That leads to that the carrier spin at the Dirac point is free to align with the external magnetic fields, and the free-aligned carrier spin is speculated to be the source of the paramagnetic susceptibility at low magnetic fields. Based on this theoretical description, the critical factor of the paramagnetic susceptibility is the existence of the free-aligned carrier spin at the Dirac point. It is known that the Fermi level location is sensitive to material components and fabrication conditions in topological materials. However, no one supports the existence of the Dirac point in all previous reports [1–5]. Without this critical evidence, their speculation and conclusion are doubted and need further examination.

To further examine this characteristic of topological materials, the magnetization characteristic of Bi_0.3_Sb_1.7_Te_3_ topological insulator single crystal was observed. Our experimental result shows the singularity paramagnetic peak in the susceptibility at low magnetic fields, which is the same as the previous reports. However, the angle-resolved photoemission spectroscopy (ARPES) revealed that the Fermi level lies below the Dirac point. The detected Fermi wavevector from ARPES is the same as the extracted value from the de Haas–van Alphen (dHvA) oscillation at high magnetic fields. These results indicate that there should be no carrier transport characteristic contribution from the Dirac point on the paramagnetic susceptibility. These results strongly support that the observed singularity paramagnetic susceptibility should not be dominated by free-aligned carrier spin texture at the Dirac point that completely violates the speculation in previous reports [1–5].

## Experimental Method

Single crystals of Bi_0.3_Sb_1.7_Te_3_ were grown using a homemade resistance-heated floating zone furnace (RHFZ). The raw materials used to make the Bi_0.3_Sb_1.7_Te_3_ crystals were mixed according to the stoichiometric ratio. At first, the stoichiometric mixtures of high purity elements Bi (99.995%), Sb (99.995%) and Te (99.995%) were melted at 700–800 °C for 20 h and slowly cooled to room temperature in an evacuated quartz tube. The resultant material was then used as a feeding rod for the RHFZ experiment. Our previous work demonstrated that topological insulators with extremely high uniformity can be obtained using the RHFZ method [10–13].

XRD pattern was measured using a D8-Discover designed by Bruker (15°–70°, per 0.01° one point). The X-ray generator voltage is 40 kV, and the current is 40 mA. Cu *α* mediated by Cu target was used in our measurement, which radiation wavelength is 1.5406 Å. Our sample would not fluoresce under K*α* beam in low angle and create polychromatic radiation. As the result, Cu K*α* beam has higher intensity than Cu K*β* which is suitable for our sample.

The magnetization measurement was performed using the commercial apparatus (Quantum design, SQUID) with a magnetic field of up to 7 T. The sample geometric sizes are 1.2 mm (length), 0.2 mm (width) and 0.2 mm (thickness). This crystal size is suitable for the SQUID measurement in three different planes. The single crystal is fixed in a specific capsule for the SQUID measurement. The capsule is mounted onto a sample rod and inserted to the magnetic field center for magnetization measurement. The sample space is continuously pumping by mechanic pump during the measurement to keep the sample space at the vacuum condition of $1\times 10^{-2}$ torr.

## Samples Characterization

**Fig. 1:**
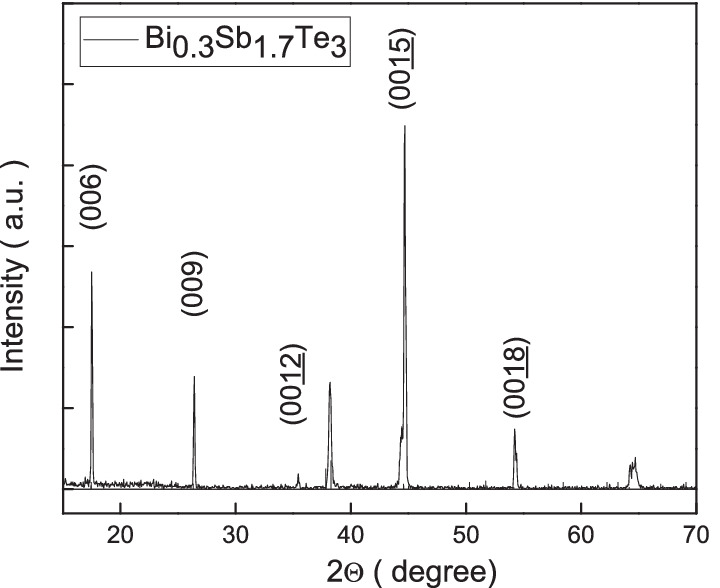
The XRD spectrum of the Bi_0.3_Sb_1.7_Te_3_ single crystal. It reveals sharp peaks that support the highly single crystallized structure

X-ray diffraction of the single crystal is shown in Fig. 1, and it shows the sharp peaks which support a highly single crystallized structure. An electron probe micro-analyzer is used to analyze the element component of the single crystal. Table 1 lists the element ratio at different zones of the grown single crystal. It reveals the uniform element ratio distribution in the single crystal. Energy-dispersive X-ray spectroscopy (EDS) confirmed that the crystal contained Bi/Sb/Te = 0.3:1.7:3.

**Table 1:** List of the element ratio at different zones of the Bi_0.3_Sb_1.7_Te_3_ crystals

	Bi	Sb	Te	Element ratio
Zone 1	6.37	33.19	60.44	Bi$_{0.32}$Sb$_{1.65}$Te$_{3}$
Zone 2	6.08	33.08	60.84	Bi$_{0.30}$Sb$_{1.63}$Te$_{3}$
Zone 3	6.75	31.97	61.27	Bi$_{0.33}$Sb$_{1.57}$Te$_{3}$
Zone 4	6.42	32.82	60.76	Bi$_{0.32}$Sb$_{1.62}$Te$_{3}$
Zone 5	6.51	33.17	60.32	Bi$_{0.32}$Sb$_{1.65}$Te$_{3}$
Zone 6	6.35	33.06	60.60	Bi$_{0.31}$Sb$_{1.64}$Te$_{3}$
Average ratio	6.41	32.88	60.71	Bi$_{0.32}$Sb$_{1.63}$Te$_{3}$

To further identify the element components in the Bi_0.3_Sb_1.7_Te_3_ single crystal rod, the inductively coupled plasma mass spectrometry (ICP-MS) analysis was performed. The analysis reveals that there are four main elemental impurities in the Bi_0.3_Sb_1.7_Te_3_ single crystal rod. The impurity elements and the related concentration are Cr (0.04 ppm), Co (5.3 ppm), Zn (0.12 ppm) and Hg (0.26 ppm). The total concentration of magnetic elements is roughly 5 ppm. This is an extremely low concentration, and one expects it would not lead to significant ferromagnetism behaviors.

**Fig. 2:**
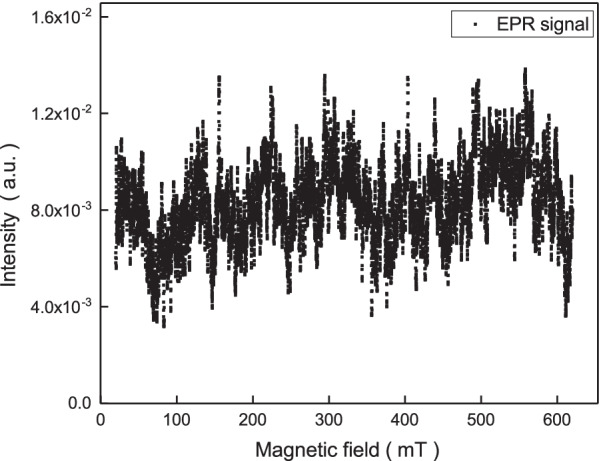
The electron paramagnetic resonance spectra as a function of magnetic fields. No obvious EPR signal of spin $S=\frac{1}{2}, 1, \frac{3}{2}$ or $\frac{5}{2}$ is observed

The room temperature X-band electron paramagnetic resonance (EPR) spectra are observed in the magnetic field range of 20 to 600 mT to characterize the intrinsic electron spin configuration. The EPR instrument model is Bruker EMX plus. As shown in Fig. 2, the EPR spectra reveal a fluctuation signal at the measured magnetic field range, and no obvious EPR signal of spins $S=\frac{1}{2}, 1, \frac{3}{2}$ or $\frac{5}{2}$. This indicates that the magnetic element concentration should be low and consistent with the results of the ICP-MS analysis.

## Results and Discussion

**Fig. 3:**
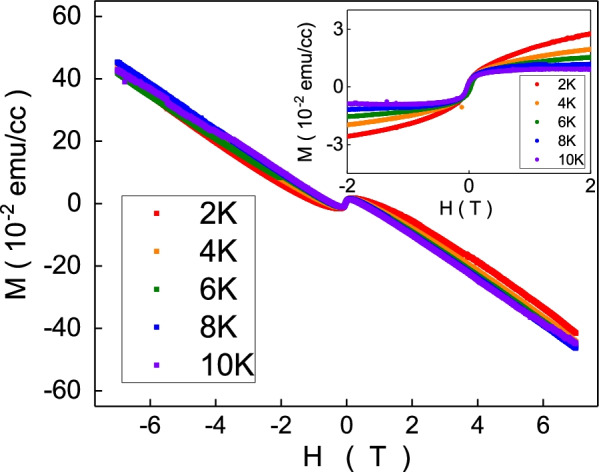
The magnetization moment as a function of magnetic fields. It is paramagnetic at low magnetic fields and diamagnetic at high magnetic fields. The inset shows the magnetization moment after subtracting the diamagnetism contribution as a function of magnetic fields

The magnetization was measured on the as-grown Bi_0.3_Sb_1.7_Te_3_ single crystal rod. Figure 3 exhibits a paramagnetic to diamagnetic crossover transition as increasing magnetic field. The transition magnetic field is roughly 2000 Oe. Theoretically, this diamagnetism originates from unpaired electron spins, and it exists in materials. The diamagnetism is usually weaker than ferromagnetism or paramagnetism at low magnetic fields. The diamagnetism is negatively proportional to the magnetic fields, and the slope is insensitive to temperatures in our experimental results. The slope is $-\,2.15\times 10^{-4}$ emu/cc Oe and is consistent with reported values in various kinds of topological insulator. Figure 3 inset shows the magnetic moment without the diamagnetism contribution.

**Fig. 4:**
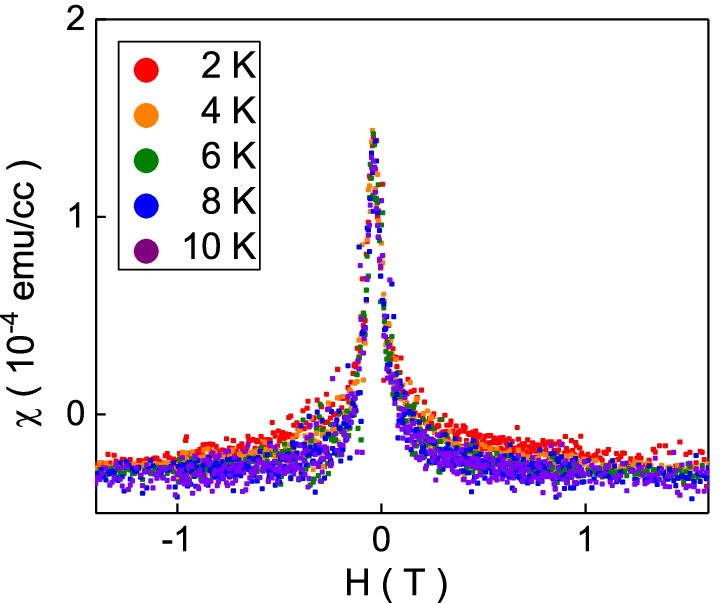
The susceptibility as a function of magnetic fields from 2 to 10 K. All data collapse onto a single curve

The susceptibility curve is obtained after taking the derivative of magnetization with respect to the external magnetic field. Figure 4 shows a paramagnetic peak, and the obtained susceptibility data points collapse onto a single curve at measured temperatures. This characteristic is the same as the recent experimental reports in various kinds of topological materials. It is understood as the free-aligned carrier spin of the surface state at Dirac point. The surface state of 3D topological insulators could be formulated using the Dirac-type Hamiltonian, $H(k_{x}, k_{y}) = \hbar v_\mathrm{F}(\sigma ^{x} k_{y} - \sigma ^{y} k_{x} )$, where $\mathbf {\sigma }$ and ${\mathbf {k}}$ are the Pauli matrix and translation momentum [14, 15]. That links the carrier spin to the carrier momentum, leading to the spin momentum locking [8, 9]. The surface state carrier possesses a particular spin helicity, and the spin momentum is perpendicular to the carrier momentum. The related spin texture at the upper and lower Dirac cone is different in clockwise direction. This particular spin texture leads to that the carrier at the Dirac point does not have any preferable orientation and free to align along with the external magnetic field. These freely oriented spins at the Dirac point are predicted to generate a paramagnetic peak in the susceptibility curve. This model was widely used to qualitatively explain the paramagnetic behavior at low magnetic fields in several topological materials [1–5]. On the basis of this model, the critical speculation of the paramagnetism is the free-aligned carrier spin at Dirac point. This singular paramagnetic characteristics will not exist in a system without a free-aligned carrier spin texture at the Dirac point. However, the previous reports never support the existence of the Dirac point in their system.

**Fig. 5:**
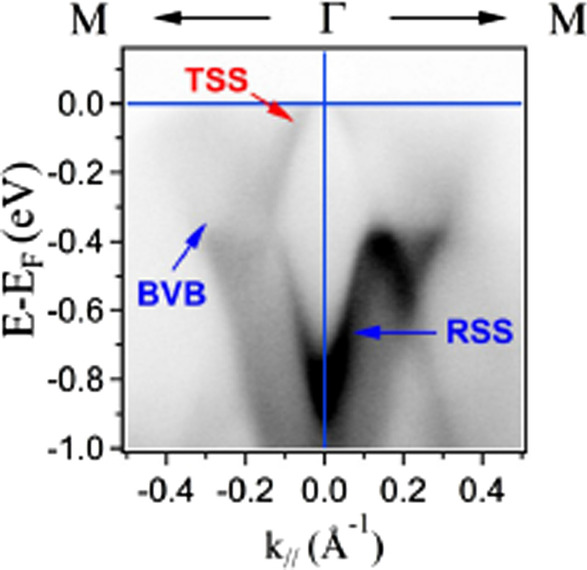
Band mapping of Bi_0.3_Sb_1.7_Te_3_ in a wide energy range along $\Gamma$–M direction using photon energy 24 eV. The Dirac point can be determined at 80 meV above the Fermi level with the extrapolation of the linear dispersion of topological surface state

To identify the topological surface state and the position of Dirac point. ARPES was used to measure the band structure of the Bi_0.3_Sb_1.7_Te_3_ single crystal. ARPES experiment was performed at TLS-BL21B1 beamline, NSRRC, Taiwan. All photoemission spectra were collected at 85 K with a base pressure $6.8\times 10^{-11}$ torr, and the energy resolution is 12 meV. Figure 5 displays the band mapping result of the Bi_0.3_Sb_1.7_Te_3_, which was measured along the $\Gamma$–M direction and recorded at photon energy 24 eV. In Fig. 5, a bulk valence band (BVB) is crossed the Fermi level, which behaves Bi_0.3_Sb_1.7_Te_3_ as a *p*-type semiconductor and consistent with previous study [16]. With the examination of the photon energy-dependent experiment, a resonant surface state disperses between − 0.4 and − 0.8 eV, which was similar to that observed in Sb$_{2}$Te$_{3}$ [17]. The band mapping result implies that the valence band of the Bi_0.3_Sb_1.7_Te_3_ is close to Sb$_{2}$Te$_{3}$ owing to less bismuth content. The topological surface state appears at binding energy 0.4 eV and disperses linearly toward the Fermi level with a crossing Fermi vector *k*_F_ = 0.04 Å^−1^ extracted from the momentum distribution curves (MDC). With the extrapolation of the linear dispersion of the topological surface state, the estimation of the Dirac point is located at 80 meV above the Fermi level. That indicates that there should be a negligible contribution from the free-aligned carrier spin texture at the Dirac point on the observed magnetization.

**Fig. 6:**
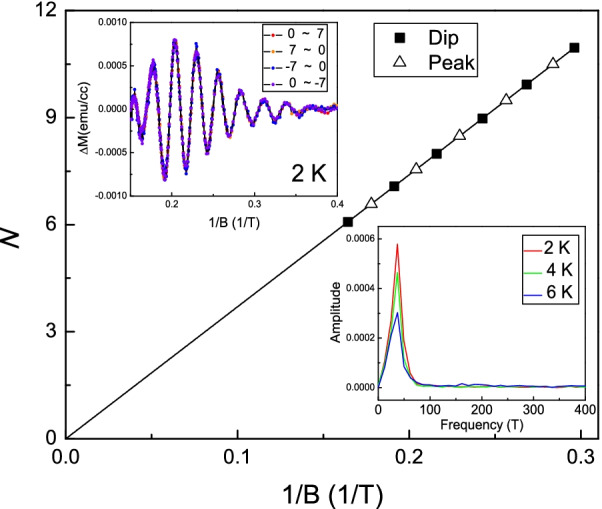
Top-left inset shows the extracted magnetization as a function of inverse magnetic fields at different sweeping directions. It shows periodic oscillations. Bottom-right inset shows the fast Fourier transform of the magnetization oscillation. It shows single oscillation peak at measured temperatures. The Landau-level fan diagram. The intercept is 0 which indicates the oscillation originates from the 2D Fermi surface

To further specify carrier characteristics of the Fermi level, quantum magnetization oscillations, dHvA oscillations, at high magnetic fields were observed. The top-left inset of Fig. 6 shows the extracted magnetization as a function of inverse magnetic fields in different sweeping directions at 2 K. It reveals periodic oscillations, and all data points in different sweeping directions collapse onto a single oscillation curve. This supports the transport characteristic uniformity and no magnetic impurities in our samples. The bottom-right inset of Fig. 6 shows the fast Fourier transform (FFT) of the measured dHvA oscillations. It shows a single sharp peak at 37 T at measured temperatures. Following the Onsager relation, $F=\frac{\hbar k^{2}_\mathrm{F}}{2\pi }$, one could estimate the $k_\mathrm{F}\propto 0.034$ Å^−1^ that is consistent with the detected value, 0.04 Å, from the ARPES. These results support that the observed magnetization oscillation is the dHvA oscillations from the topological insulator surface state. The dHvA magnetization oscillation could be expressed as:

$$\begin{aligned} \Delta M \propto \sin [2\pi (F/B - \gamma )]. \end{aligned}$$

where *F* is the oscillation frequency, *B* is the magnetic field, and $\gamma$ is expressed as $\gamma = 1/2 - \beta + \delta$. The $\beta$, Berry phase, is 1/2 for Dirac-type system with linear E–K dispersion and 0 for the system with parabolic band structure. The $\delta$ is determined by the dimensionality, and it would be 0, 1/8 or $-\,1/8$ in the 2D Fermi surface, 3D Fermi surface with hole carrier or 3D Fermi surface with electron carrier, respectively [18]. As shown in Fig. 5, the ARPES shows the surface state with linear E–K dispersion. That supports the $\beta$ is 1/2. As shown in Fig. 6, the linear fitting reveals that the intercept is 0, which indicates the $\delta$ is 0. This is consistent with the characteristic of the 2D surface state in 3D topological insulators. These results confirm that dHvA originates from the surface state of the 3D topological insulator, and the Fermi level lies below the Dirac point in the Bi_0.3_Sb_1.7_Te_3_ single crystal.

The theoretical calculation supports that the susceptibility could be expressed as

$$\begin{aligned} \chi (B) \cong \frac{\mu _{0}}{4 \pi ^{2}}[\frac{(g\mu _{B})^{2}}{\hbar \nu _\mathrm{F}}\Lambda - \frac{2(g\mu _{B})^{3}}{\hbar ^{2} \nu _\mathrm{F}^{2}}|B|] \end{aligned}$$

at the zero chemical potential and temperature [1]. The *g* is the Landé *g*-factor, and $\Lambda$ is the effective size of the momentum space contributing to the singular part of the free energy. The peak height is determined by the $\Lambda$ which depends on the band structure, and the value would vary from system to system. The susceptibility peak slope near the zero magnetic fields depends on the Fermi velocity, $\nu _\mathrm{F}$. Based on the data in Fig. 4, one estimates the $\nu _\mathrm{F} \sim 6.6 \times 10^{4}$ m/s. This value is one order smaller than the estimated value from the ARPES. This result further confirms that the observed low magnetic field paramagnetic peak in the susceptibility curve does not originate from the carrier spin texture at the Dirac point.

**Fig. 7:**
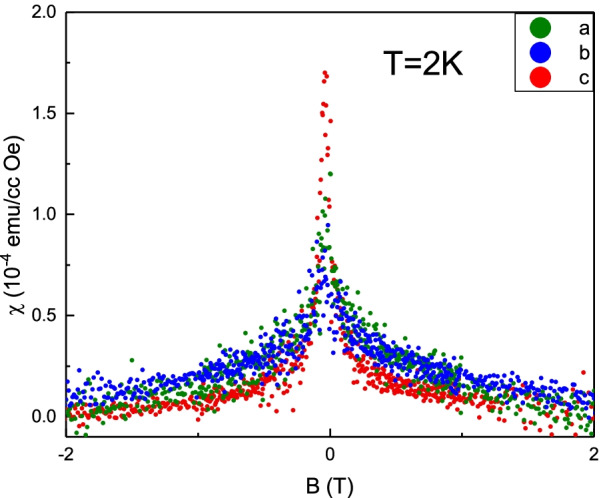
The measured magnetic field-dependent susceptibility in three orthogonal directions at 2 K. It exhibits different peak maxima at zero magnetic fields which implies that observed paramagnetic peaks should not originate from a system with randomly and uniformly distributed ferromagnetic elements

It is suspected that the observed paramagnetic peak originates from the ferromagnetic elements in our system and one does not need a new mechanism to identify the observed paramagnetic peak at zero magnetic fields. It might originate from magnetic elements that penetrate into our system during the sample fabrication and experiment operation. It is expected that these unavoidable magnetic elements should be randomly and uniformly distributed and would not form a magnetic moment ordering in a specific crystal direction in the sample. The magnetic response would be insensitive to geometrical directions in a system with randomly and uniformly distributed ferromagnetic elements. Figure 7 shows the measured magnetic field-dependent susceptibility in three orthogonal directions at 2 K. It exhibits different peak maxima at zero magnetic fields, which implies that observed paramagnetic peaks should not originate from ferromagnetic elements in our system. On the other hand, the thermal energy could randomize the orientation of magnetic moment and the magnitude of magnetic moment would be sensitive to the thermal energy. The competition between the thermal energy and the coupling energy of the magnetic moment and the external magnetic field would directly influence the susceptibility response. The observed susceptibility peak corresponds nearly zero to magnetic fields. The magnetic moment would not be strongly pinned at a specific direction at such a low magnetic field, and coupling energy should be low. It is expected that thermal energy will dominate over the competition, which leads to the temperature-dependent susceptibility. Thus, the magnetic impurity-induced paramagnetic susceptibility peaks should be temperature dependent. But, the paramagnetic susceptibility peaks are temperature independent in our observation and related reports. This implies that the observed paramagnetic susceptibility peaks should not originate from the magnetic elements. Further, if the susceptibility of the paramagnetic peaks originates from the magnetic elements, it should be related to the concentration of magnetic elements. It is worthy to pay attention to the fact that the reported susceptibility of the paramagnetic peaks at zero magnetic fields is about 10$^{-7}$ emu/gOe in various kinds of topological materials [1–5]. These materials are from different growing conditions and experimental treatments. It is not expected that different fabrication processes and experimental treatments lead to similar concentrations of ferromagnetic elements. The EPMA shows no detectable amount concentration of ferromagnetic elements in our crystal. If the unavoidable ferromagnetic elements contaminate during the preparation and experimental operation, one would expect this similar pollution in the ordinary materials. But, such paramagnetic susceptibility peaks at zero magnetic fields are not observed in various kinds of materials.

On the other hand, it comes to our attention that the Rashba spin-split band structure in Bi-rich Bi_2_Se_3_ nanoplates and giant Rashba semiconductor BiTeI exhibits similar behavior [19]. Theoretically, carriers with different spin directions will have a specific trajectory in the E–K space under external magnetic fields. The carrier trajectory is equivalent to the carrier orbital motion in real space, and the different spin directions are associated with electron motions in clockwise and counterclockwise. Due to the Zeeman energy splitting, carriers occupy the different spin-direction states, and carrier orbital magnetization leads to the paramagnetic peak near the zero magnetic fields. For this carrier orbital magnetization, the paramagnetic peak is independent of the carrier spin helicity of the surface state in topological insulators, and paramagnetic peak in susceptibility is related to the location of the Fermi level. That might be the mechanism of the singularity paramagnetic peak in our system with a *p*-type surface state.

## Conclusion

The magnetization is performed in Bi_0.3_Sb_1.7_Te_3_ single crystal. It reveals a paramagnetic peak in the susceptibility curve which collapses onto single curves at temperatures. It is speculated to be originated from the free-aligned spin texture at the Dirac point. The ARPES reveals that the Fermi level lies below the Dirac point. The Fermi wavevector extracted from the dHvA oscillation is consistent with the result in the ARPES. Our experimental results support that the observed paramagnetic peak in the susceptibility curve should not originate from the free-aligned spin texture at the Dirac point.

## Data Availability

The datasets generated during and/or analyzed during the current study are available from the corresponding authors on reasonable request.

## Abbreviations

EDS: Energy-dispersive X-ray spectroscopy
EPR: Electron paramagnetic resonance
XPS: X-ray photoelectron spectroscopy
ARPES: Angle-resolved photoemission spectroscopy
dHvA: De Haas–van Alphen
ICP-MS: Inductively coupled plasma mass spectrometry
